# Microplastics in Mediterranean Sea: A protocol to robustly assess contamination characteristics

**DOI:** 10.1371/journal.pone.0212088

**Published:** 2019-02-11

**Authors:** Mikaël Kedzierski, Jonathan Villain, Mathilde Falcou-Préfol, Marie Emmanuelle Kerros, Maryvonne Henry, Maria Luiza Pedrotti, Stéphane Bruzaud

**Affiliations:** 1 Institut de Recherche Dupuy de Lôme, Université Bretagne Sud, Unité Mixte de Recherche Centre National de la Recherche Scientifique, Lorient, France; 2 Unité de Recherche Informatique et Automatique, Institut Mines-Télécom Lille Douai, Université Lille, Lille, France; 3 Laboratoire d'Océanographie de Villefranche-sur-Mer, Sorbonne Universités, Unité Mixte de Recherche Centre National de la Recherche Scientifique, Villefranche sur mer, France; 4 LER PAC, IFREMER, La Seine-sur-Mer, France; VIT University, INDIA

## Abstract

The study of microplastic pollution involves multidisciplinary analyses on a large number of microplastics. Therefore, providing an overview of plastic pollution is time consuming and, despite high throughput analyses, remains a major challenge. The objective of this study is to propose a protocol to determine how many microplastics must be analyzed to give a representative view of the particle size distribution and chemical nature, and calculate the associated margin error. Based on microplastic data from Tara Mediterranean campaign, this approach is explained through different examples. In this particular case, the results show that only 3% of the collected microplastics need to be analyzed to give a precise view on the scale of the North West Mediterranean Basin (error <5%), and 17.7% to give an overview manta per manta (error <10%). This approach could be an important practical contribution to microplastic studies.

## Introduction

The contamination of the marine environment by plastics is a major environmental concern and has been the subject of an increasing number of studies and surveys since 2000 [1]. In this context, Tara Expedition, a French non-profit organization acting for the environment since 2003, conducted microplastic sampling for 7 months in 2014 across the Mediterranean Sea. The objective of this expedition was to characterize microplastic contamination in order to better describe the effects of plastic waste on marine ecosystems. In particular, the Tara Mediterranean Consortium is trying to evaluate the spatial distribution of floating debris in the Mediterranean Sea, to chemically characterize the different types of plastics and to characterize bacteria communities [2] as well as the micro- and macro-organisms fixed on plastic particles. In the long term, the scientific knowledge provided by this project will make it possible to better target the actions to be taken to reduce pollution by plastic waste in the Mediterranean Sea.

More broadly, the number of analyses proposed to properly characterize the contamination of the natural environment by microplastics has increased and diversified in recent years [3]. Thus, when dealing with baseline observations such as particle numbers [4–6], color identification [7,8], particle size [9,10] or particle categorization (e.g. fibers, fragments) [7,8], more complex descriptors related to surface morphology [8,11] or the determination of the polymer nature [12–15] are now more commonly used. Contamination by pollutants such as heavy metals [11,16,17], endocrine disruptors [11] and Persistent Organic Pollutants [8,18–20] is increasingly being studied. In addition to these physico-chemical analyses, biological parameters such as the organic communities living on the surface of the microplastics are beginning to be analyzed [2,21–23]. Different analyses can also be made on the same sample, such as the measure of the microplastic and the plastic chemical composition determination [24]. This can imply that destructive analyses, which can be considered as a limitation in the study of microplastics [25], should be restricted to a minimum and libraries of microplastics should be set up and organized to be exploited over a long period of time. However, the analysis of microplastics can be complex, even impossible, when their number is too large. Microplastic pollution, although ubiquitous, is not uniformly distributed over the planet. Areas with higher contamination concentrations have been identified such as the five oceanic gyres [26–29], the Arctic [9], the China Sea [30,31] and the Mediterranean Sea [24,30–32] and a very large number of microplastics have been collected in these areas. In such case, it is inconceivable to carry out the different analyses of all the collected microplastics. It is therefore necessary to select only a representative part. However, reducing the number of analyzed microplastic samples leads to a decrease in sample representativeness.

In 2014, the Tara Mediterranean expedition collected tens of thousands of microplastics from various sites on the surface of the Mediterranean basin using a manta net. Some of these samples were fully analyzed and two types of parameters were particularly studied as part of our study: the granulometric distribution of the microplastics and their chemical nature. The objective of this study is to verify whether it would have been possible to reduce the amount of work by subsampling these microplastics. To help solve the problem of the analysis of a large population of microplastics, a new analytical approach is proposed, based on a statistical sub-sampling method commonly used in surveys. The originality of this work is the use of this statistical sub-sampling method in the study of microplastics. This approach, which does not depend on the parameters to study or the analytical method, will be tested on the data sets of both parameters (granulometric distribution, chemical nature). The values of these parameters will be calculated and compared with the values obtained by statistical sub-sampling. This will make it possible to verify whether this approach can be applied in the particular case of the study of microplastics. In order to make this article as didactic as possible, it will be organized according to the following plan: (1) First, we will present the sample collection protocol, sample preparation for analysis and analysis protocol. (2) We will then explain the statistical protocol used to estimate the proportions of the two parameters followed. (3) We will finally apply our protocol to a real application: the study of the contamination of the North-West basin of the Mediterranean Sea by microplastics.

### Sample collection, preparation and analysis

The sampling campaign was conducted in the Mediterranean Sea from May to November 2014 in the framework of the TARA Mediterranean Expedition. The aim of the project is to describe the impacts of plastic waste on marine ecosystems of the Mediterranean Sea.

### Sample collection

**Fig 1:**
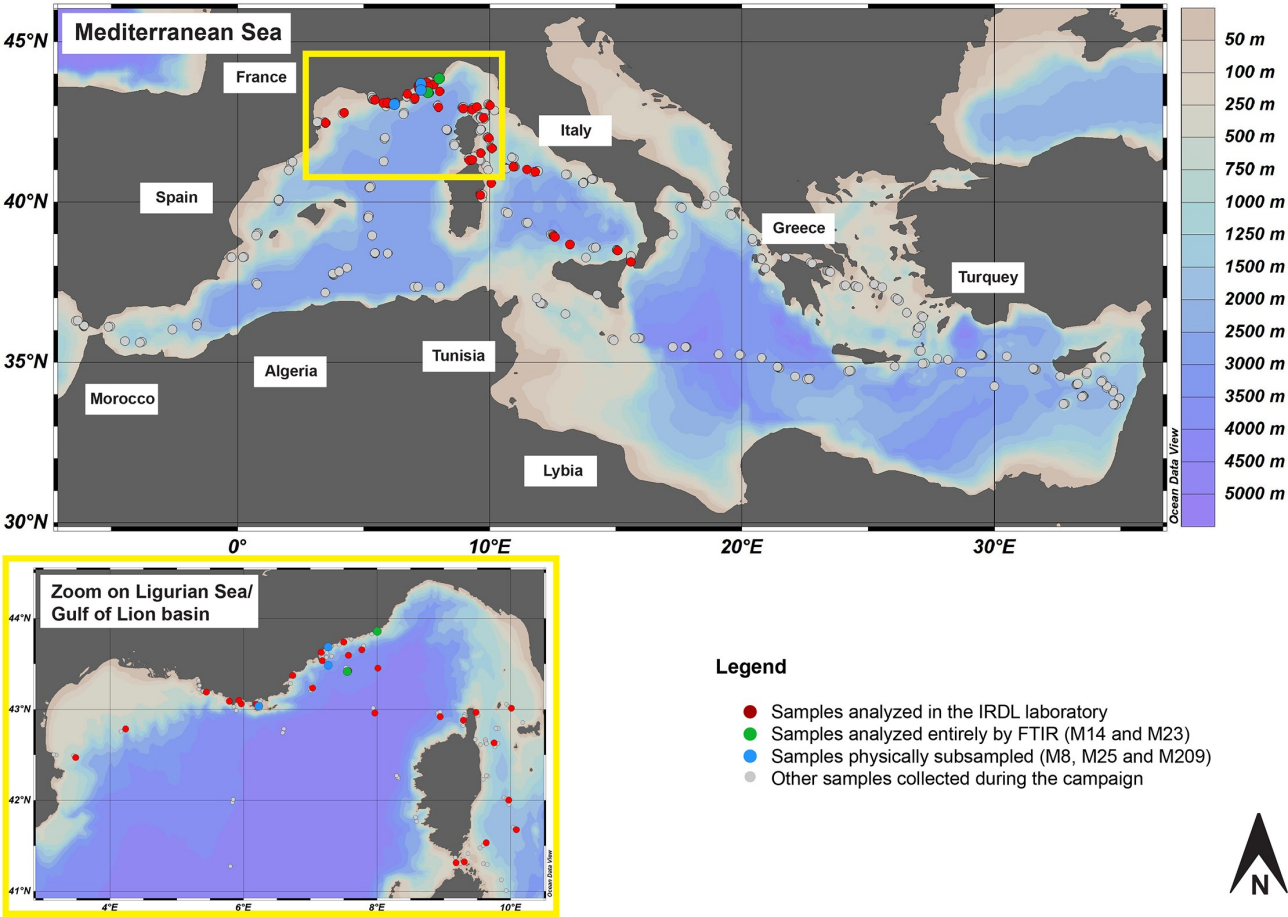
Sampling effort during the Tara campaign in the Mediterranean Sea. Ocean Data View [33].

Microplastics were sampled using a 4.4-m-long manta net of 333 μm mesh size with a net opening of 16 cm x 60 cm by trawling on 120 sites by day and by night (Fig 1). The manta towed at the sea surface for ca. 60 min behind the boat at an average speed of 2.5 knots. Consequently, the average filtered volume of each manta trawl was about 507 m^3^.

The physical-chemical data were collected using a thermosalinograph (TSG) and a hyperspectral spectrophotometer (AC-S). Ocean Color satellite images were supplied by ACRI-ST and the Mercator circulation model was used to determine the areas of interest for the sampling. Geographical coordinates are available at Pangea Data Publisher http://www.pangaea.de.

### Preparation of the samples

Preserved organic material was first removed from microplastics under a dissecting microscope. Particles were counted and measured using the ZooScan image analysis method at the Laboratoire d’Océanographie de Villefranche-sur-Mer (LOV, Villefranche-sur-Mer, France) [24] and then checked and weighed at IFREMER, Laboratoire LER/PAC. Finally, the microplastics from 77 manta trawls were transferred to the Institut de Recherche Dupuy de Lôme (IRDL, Lorient, France) for analysis of their chemical nature. To test the statistical sub-sampling protocol, the particle size range of microplastics from 42 mantas trawls was used.

Contamination risks were avoided during the sample preparation stage by cleaning the different parts of the apparatus with distilled water, ethanol and/or acetone, and by working in controlled conditions (chemical laboratory). The preparation step and the oven step were performed in an area dedicated to the treatment of Tara Mediterranean Sea samples. Despite the need to handle the analyses of the microplastics in the open-air, the time for interaction of the collected microplastics with potential airborne microplastics was minimized as far as possible. After each analysis of microplastics by Attenuated Total Reflectance Fourrier Transform Infrared Spectroscopy (ATR-FTIR), the sample holder was cleaned with ethanol or acetone. The sample chamber was also cleaned out with a vacuum cleaner after every sixty analyses. Furthermore, the use of plastic apparatus was avoided as far as possible. If this was impossible, the Fourier-transform infrared spectroscopy (FTIR) spectra from these materials were obtained to check whether potential contamination had occurred.

The sample preparation protocol used here is partially based on classic protocols [34–37], but also includes an original step which involves the isolation and naming of each plastic particle items in order to build up a microplastics library. For each of the 42 mantas, the first step was wet sieving to separate the microplastics according to their size class. A stainless-steel sieving cascade with ten different mesh sizes ranging from 5 mm to 32 μm was used to separate the items into ten size classes (>5 mm, ]5–4] mm, ]4–2] mm, ]2–1] mm, ]1–0.5] mm, ]0.5–0.315] mm, ]0.315–0.250] mm, ]0.250–0.125] mm, ]0.125–0.063] mm and ]0.063–0.032] mm). The 5 mm sieve retains particles in the size class coarser than 5 mm. Four sieves with mesh sizes smaller than 0.315 mm were added to retain fibers and small microplastic particles that could be initially present in particle aggregates. Wet sieving was carried out with 5 L of distilled water using a slow flow rate (around 0.04 L.s^-1^) through the sieve set. Then, the microplastics collected in each size class were placed in glass Petri dishes and left for around 24 hours in an oven at 50°C. The particles belonging to size classes coarser than 0.315 mm were then transferred to 96-well microplates identifier (i.e.: TM0001A1 for “*Tara Méditerranée*”, microplate n°0001 and well n°A1). Each of these microplastics was thus isolated and was named with a unique identifier. Owing to this procedure, the size class of each particle was noted. Particles finer than 0.315 mm were stored in chromatography tubes and not included in this study. Basic information such as the unique identifier, the size range, the microplastic color, and any further observations were noted to provide input for the project database. Thus, the microplastic isolation step allows us to give, for every particle present on the microplates, a unique identifier. This code is necessary for the random draw step.

### Fourier-transform infrared spectroscopy (FTIR) analysis

The chemical nature of plastic items in manta M23 (207 microplastic particles) and M14 (767 microplastic particles) was determined to use non-destructive analysis using an Attenuated Total Reflection Fourier Transform Infrared spectrometer (ATR-FTIR Vertex70v, Bruker). These two mantas were chosen since the number of microplastic particles collected is representative of the medium (100 to 500 items) and large samples (more than 500 items). All spectra were recorded in the absorbance mode in the 4000–600 cm^-1^ region with 4 cm^-1^ resolution and 16 scans. To obtain a better quality data, each plastic fragment was placed on the germanium diamond cell (ATR Golden Gate) mounted on the FTIR spectrometer. The spectrum so obtained was then compared with reference spectra to identify the chemical nature of the collected fragment [24].

### Statistical approach and protocol

This protocol is based on a classic statistical approach from survey studies. In the following, a statistical approach and then, an estimation protocol will be presented. A detailed version of the statistical protocol is available in the supplementary materials (S1 File).

### Statistical approach

In a population (in this study, microplastics of the Mediterranean Sea), to estimate an unknown proportion π∈[0;1] of units possessing a characteristic *C* (e.g. the chemical nature of a microplastic), an estimation of the parameter of a Bernouilli law is used. In this configuration, it is considered that each microplastic is randomly drawn and therefore

P(themicroplasticpossessthecharacteristicC)=π

or

P(themicroplasticdoesnotpossessthecharacteristicC)=1−π.

#### Proportion estimator

The random variable X is defined by *X* = 1 if the chosen unit has *C* and *X* = 0 otherwise. Knowing the distribution law of π, it was possible to construct a good estimator p for the proportion π from a sample of size n. So, an estimation p corresponds to *E*[*X*] and could be calculated by

$$p=\frac{1}{n}\sum_{i=1}^{n}x_{i}$$ (1)

For this estimator, since the distribution law of X is known, it was possible to construct a confidence interval for p. To construct this interval, two configurations had to be denoted. The first configuration, in which the global population size (N) is unknown, was used in the case of the Mediterranean Sea, since it is not possible to know the exact number of microplastics present. The second configuration was the case in which the population size (N) is known. The number (N) of microplastics collected manta by manta was known and the proportion would be estimated from a subsample of size (n). Based on these two configurations, a confidence interval could be given for each one.

#### Confidence interval

Once the estimator p of the proportion had been determined, the associated confidence interval had to be calculated. In the configuration where N is unknown, the confidence interval for the confidence level α is given by

$$IC_{\frac{\alpha }{2}}=\left[{p\pm u}_{1-\frac{\alpha }{2}}\sqrt{\frac{p(1-p)}{n}}\right]$$ (2)

with $u_{1-\frac{\alpha }{2}}$ the fractal of order *α* of the standardized normal law. It is common to take as degree of confidence of 95% (*i*.*e*. *α* = 0.05; $u_{1-\frac{\alpha }{2}}=1.96$). For the configuration in which the proportion was estimated when N is known, a confidence interval for confidence level α was given by

$${IC}_{\frac{\alpha }{2}}=\left[{p\pm u}_{1-\frac{\alpha }{2}}\sqrt{\frac{p(1-p)}{n}*\frac{\left(N-n\right)}{\left(N-1\right)}}\right]$$ (3)

with $u_{1-\frac{\alpha }{2}}$ the fractal of order *α* of the standardized normal law. The protocol presented below is based on this statistical approach.

#### Protocols

To estimate the proportion of microplastics with a characteristic C, two protocols corresponding to the two configurations were used, following the 4 steps described below. The first step was the estimation of the number of microplastics needed to obtain a given accuracy (*i*.*e*. the value measuring half width of the confidence interval). The second one was the estimation of the proportion. The third step consisted in giving the confidence interval of the estimated proportion. The last step described the tests used for comparing two proportions.

#### Step 1: Number of microplastics

To be able to study the characteristics of the collected microplastics, it is first necessary to determine the minimum number of particles to be studied to reach a certain confidence level in the estimated proportion. In the statistical approach (3.1), it had been shown that accuracy depended on the proportion (p) and size of the sub-sample (n). In the first configuration, the difficulty was that p is unknown. Nevertheless, an upper bound can be found to the function *p*(1−*p*) as it is maximum when $p=\frac{1}{2}$.

In the configuration in which the estimation of the proportion of microplastics possessing a characteristic C in the Mediterranean Sea was aimed at p can therefore be substituted by $\frac{1}{2}$. Then, the number of microplastics that had to be randomly drawn was calculated for three classic values of accuracy: 5%, 2.5% and 1%. To this end, the following equation was used:

$$n=\frac{{{(u}_{1-\frac{\alpha }{2}})}^{2}}{4\varepsilon^{2}}$$ (4)

In the configuration in which the aim was to estimate the proportion of microplastics possessing a characteristic C in a manta, N is known. For a given error, the number of microplastics in the sampled population was obtained as follows:

$$n=\frac{\frac{1}{4}+\frac{\varepsilon^{2}}{{\left(u_{1-\frac{\alpha }{2}}\right)}^{2}}}{\frac{\varepsilon^{2}}{{\left(u_{1-\frac{\alpha }{2}}\right)}^{2}}+\frac{1}{4N}}$$ (5)

From the Eqs (4) and (5), it was then possible to calculate the number of microplastics to analyze for a given accuracy.

#### Step 2–3: Proportion and confidence interval

Once the minimum number of particles to be analyzed had been determined, microplastics can be subsampled and analyzed. The proportion of microplastics with the characteristic C had to be calculated using Eq (1). Then, the confidence interval of order α was calculated. To construct the confidence interval, the Eq (2) was used for the first configuration (Mediterranean Sea) and the Eq (3) for the second configuration (estimation manta by manta).

#### Step 4: Comparison of the proportions

Now that the proportions were estimated and the confidence intervals defined. The proportions (p) could be compared with a theoretical value or with the proportion of a different sample. So, to assess the significance of the difference between the values of the proportions (p) obtained after sub-sampling and those of references (π), two statistical indicators are calculated: the absolute error and the test of equal or given proportions. To compare the estimated proportions on two independent samples, the *χ*^2^ test is used because of the normal distribution of the proportions.

### Calculation tools

This protocol was applied to study the proportions of the size and chemical parameters of the microplastics collected in the Mediterranean Sea.

The subsampling was performed using R software version 3.1.2 (R Core Team, 2014) using the package “base” version 3.1.2 and the “sample()” function (Becker et al., 1988; Ripley, 1987) and the proportion test between the full population and the subsample population were performed using the package “stats” version 3.1.2 and the “prop.test ()” function (Newcombe, 1998a; Newcombe, 1998b; Wilson, 1927).

### The protocol in a Nutshell

The method proposed in the publication can be summarized in 5 fundamental steps: (1) Manta after manta, the particles are placed in the wells of microplates. Each particle then receives a unique identifier. (2) The user determines whether he wants to study the contamination characteristics for each of the mantas or for the area covered by several mantas. He will then choose the equation system corresponding to his needs. (3) The user thus determines the accuracy of the results acquired for his study. From this information, it determines the number of particles to be analyzed using equations. (4) A list of identifiers containing the required number is randomly drawn, using the R software in this study, but spreadsheet software can easily perform the same operation. (5) The particles are analyzed according to the method desired by the user.

## Results and discussion: Application to the Mediterranean Sea

Tara Mediterranean expedition collected tens of thousands of microplastics on the surface of the Mediterranean basin that now need to be analyzed based on the protocol developed in this article. First, the proportions of the different size classes will be estimated at the scale of the North-West basin of the Mediterranean Sea. Then, proportions of the different size classes will be estimated manta by manta. Finally, the protocol will be used to study the proportions of the different types of polymers that can be observed in the samples.

### Mediterranean Sea

#### Step 1: Number of microplastics

**Fig 2:**
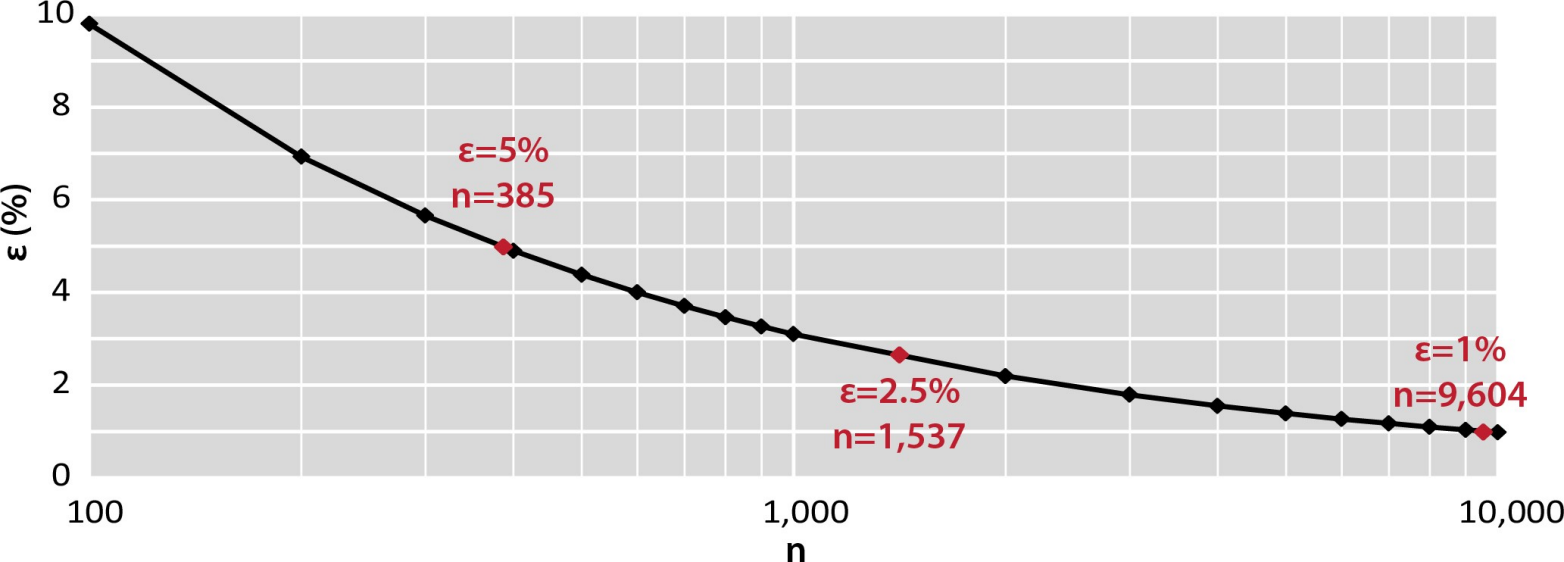
Statistical subsampling of the full population. Theoretical evolution of the accuracy (ε; %) as a function of the population randomly drawn (n). When n reached 385 microplastics, ε is lower than 5%.

The accuracy (ε) was calculated as a function of the number of random drawn particles (n ∈ [10^2^; 10^4^]). So, the n which for ε is equal to 5% is determined. The accuracy values do not decrease linearly when the number of particles randomly drawn (n) increases (Fig 2). The accuracy increases rapidly before n = 2,000 particles. Then, the increase in the number of particles drawn at random beyond 2,000–3,000 particles does not significantly improve the quality of the results compared to the required analytical effort. Thus, calculations show that 385 microplastics randomly drawn are sufficient to determine the proportions with the accuracy of 5%. With 1,537 microplastics the accuracy of the results increases up to 2.5% and up to 1% with 9,604 microplastics. In the case of this study, the proportions of the different size classes will be calculated for these different accuracies in order to show the impact of the choice of ε on the results. In the case of a real study, a single accuracy value is sufficient.

#### Steps 2–3: Proportion and confidence interval

**Fig 3:**
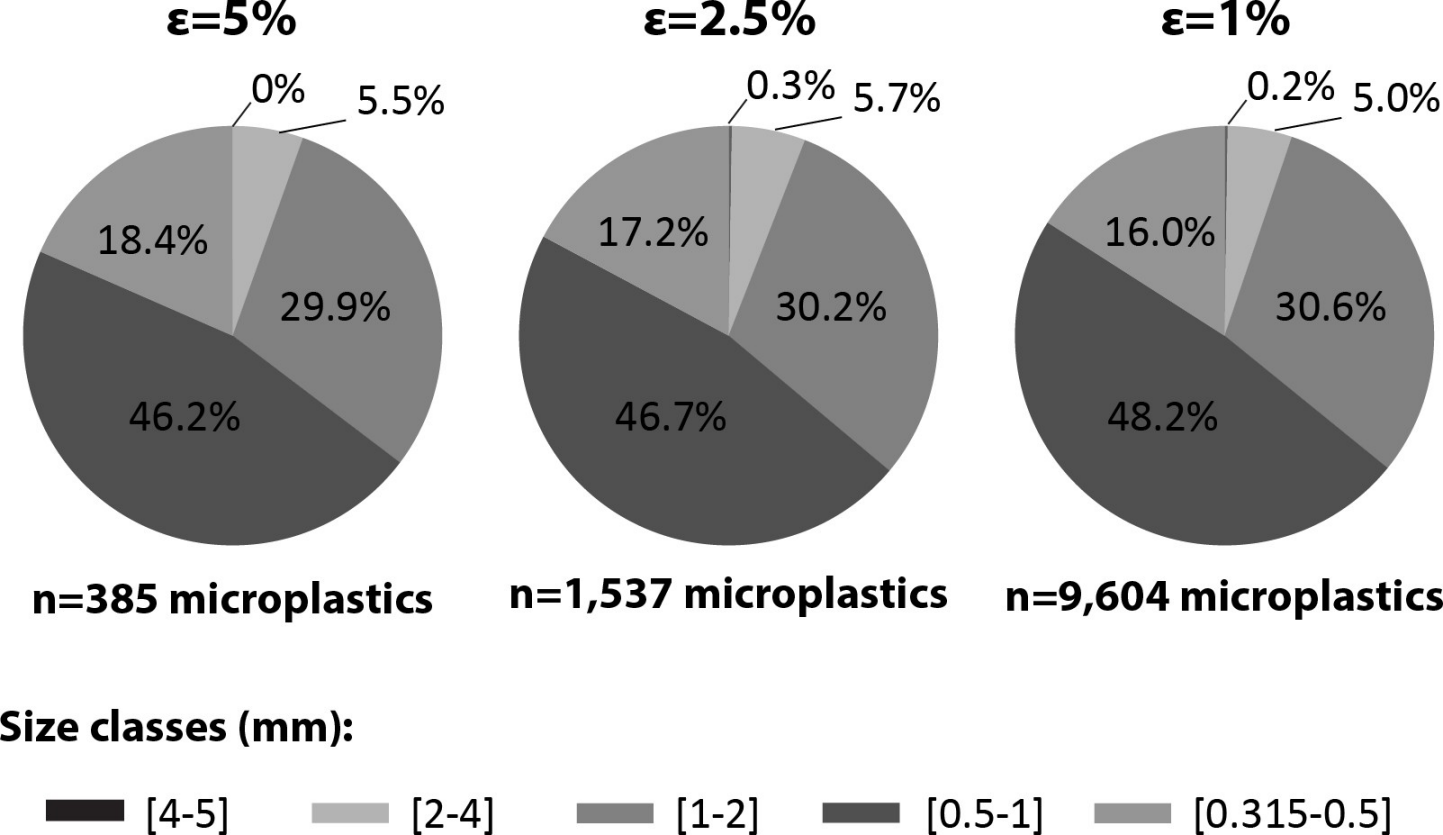
Results of the proportion of the class sizes for different accuracy values. Despite the significant increase in the number of particles analyzed, the proportions are very similar.

For the accuracy of 5%, 385 microplastics were randomly subsampled. The results show that the size distribution of microplastics is dominated by the size class [0.5–1] mm, which accounts for 46.2% of the particles (Fig 3). Next, the size classes [1–2] mm and [0.315–0.5] mm are two intermediate size classes with 29.9% and 18.4% of the microplastics respectively. The size class [2–4] mm is less observed with only 5.5%. The size class [4–5] mm is not observed. For the accuracy of 2.5%, 1,537 microplastics were randomly subsampled. The size distribution of microplastics is also dominated by the size class [0.5–1] mm, which accounts for 46.7% of the particles. The size classes [1–2] mm and [0.315–0.5] mm remain here two intermediate size classes with respectively 29.9% and 18.4% of the microplastics. Size classes [2–4] mm and the [4–5] mm are less observed with only 5.7% and 0.3% of the collected microplastics, respectively. Finally, for ε = 1%, 9,604 microplastics were randomly subsampled. The same size distribution as for the two previous results is observed here. The size class [0.5–1] mm accounts for 48.2% of the particles. The size classes [1–2] mm and [0.315–0.5] mm represent 30.6% and 16.0% of the microplastics, respectively. Last, size classes [2–4] mm and the [4–5] mm are less observed with only 5.0% and 0.2% of the collected microplastics, respectively.

#### Step 4: Comparison of the proportions

The proportions obtained for the three accuracy values selected show little differences. The maximum absolute error (εa) calculated for the same proportion, but obtained for two different accuracy values, is equal to 2.5%. The mean absolute error between proportions for ε = 5% and ε = 1% is 1.2%, and 0.8% when comparing ε = 2.5% and ε = 1%. The *χ*^2^ tests show no statistical difference between the three size distributions calculated for each of the accuracy values (p-value >> 0.05). The z test values are lower than 1.96, so the null hypothesis cannot be rejected.

#### Implication for the study of large microplastic data

The statistical method applied in this study allows a substantial reduction in the number of microplastics to be analyzed. The results obtained by analyzing 385 to 9,604 randomly selected microplastics provide a representative view of the entire population, with a maximum error between 5 and 1%. These results also highlight the fact that an increase in analytical effort after 2,000–3,000 microplastics does not significantly improve the quality of the results compared to the required analytical effort. This methodology can be used in the case of large-scale sampling campaigns where the number of microplastics collected could be of the order of tens of thousands of particles. With this approach, it will be relatively easy and fast, compared to analyzing the complete data set, to obtain robust results and determine overall values for the study area. Furthermore, an increasing number of physicochemical descriptors such as Persistent Organic Pollutants [38–40], endocrine disruptors [11,41], heavy metals [11,16,42–44] are studied in plastic particles. In the same way, an increasing number of parameters are being used to study organisms living on microplastic surfaces [21,22,45,46]. It is therefore likely that, in the relatively near future, research programs will systematically attempt to describe microplastic pollution using different descriptors. This will involve multiple analyses, which will be difficult, if not impossible to perform on oversized microplastics sample populations. The application of this protocol is a very effective solution to achieve study application of multiple sample analysis including several thousand microplastics.

### The manta by manta approach: The example of the size classes

We then tested the subsampling protocol manta by manta (N = 13,115 microplastics from 42 mantas). The aim is to determine the proportions of the different size classes with an “acceptable accuracy” for each manta.

#### Step 1: Number of microplastics

**Fig 4:**
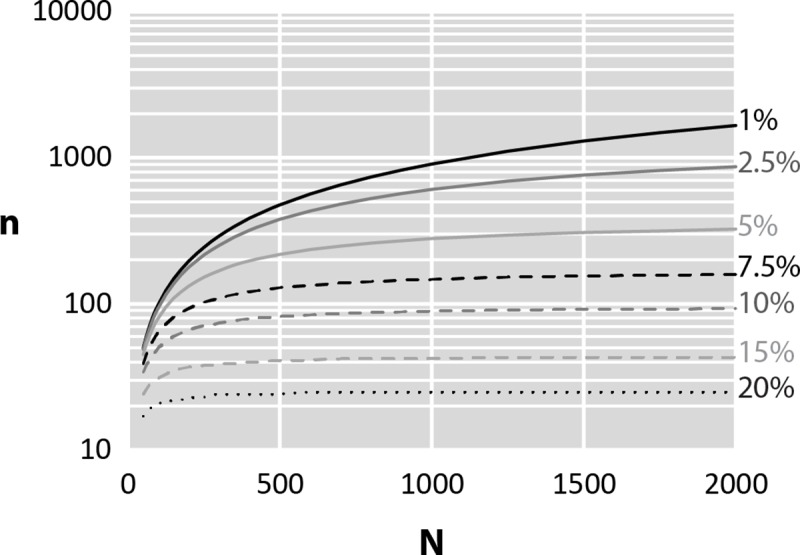
Variations in the number of particles subsampled (n) as a function of the number of microplastics collected in the manta (N) for different accuracy values.

The number of particles to be subsampled (n), for a given number of particles present in the manta (N), increases strongly when the value of the precision of the results decreases (Fig 4). Thus for N = 2,000, n = 1,656 (83%) for ε = 1% whereas n = 324 (16%) for ε = 5% and n = 93 (5%) for ε = 10%. The decrease in the number of particles is also not as significant for all N values. For example, for ε = 5% the number of particles in the manta must be equal to 400 for the n/N ratio to be less than 0.5. For ε = 10%, this ratio is reached for N = 100. Thus, under these conditions, when N tends to infinity, the number of microplastics that need to be subsampled tends to 386 for ε = 5% and 98 particles for ε = 10%. In the particular case of this study, the accuracy of 10% has been chosen regarding the number of manta and the effort required to analyze them. In fact, in the case of this study, we estimate that our capacity of analysis is limited to 2,600 particles among the 13,155 collected (20%). Using the methodology proposed on the Tara Mediterranean cruise data, the number of particles to be analyzed, calculated on the basis of the constraints established that for an accuracy of 10%, decreased from 13,155 to 2,323. This accuracy value may seem high compared to the value conventionally used in many scientific studies (5%), but it gives a relatively accurate idea of the proportions of the parameter chosen for the study. From the point of view of the present example, it allows the analysis of 42 manta traits, which, because of analytical limits, could not have been analyzed. Thus, with ε = 10%, the number of microplastics randomly selected per manta is less than 100, regardless of the number of microplastics in the sample. The impact of this approach is more or less marked depending on the number of microplastics per sample. In fact, if the number of particles is less than 100, the analytical effort remains high. Nevertheless, the gain increases rapidly with the number of selected particles. For samples containing more than 1,000 microplastics, less than 10% of particles need to be analyzed. The choice of the accuracy value is necessarily a compromise between the desire to obtain results as close as possible to the real values and the limits in terms of the analysis capacity of the samples. In this example, this analysis capacity should be increased to 3,500 microplastics to achieve the accuracy of 7.5% and to 5,500 for the accuracy of 5%. Thus, even a slight variation in the desired accuracy has important consequences on the number of samples to be analyzed.

According to this, the number of microplastics, required to obtain an arbitrary maximum error of 10%, was calculated for each manta and the proportion of the size classes was determined for each manta.

#### Steps 2–3: Proportion and confidence interval

**Fig 5:**
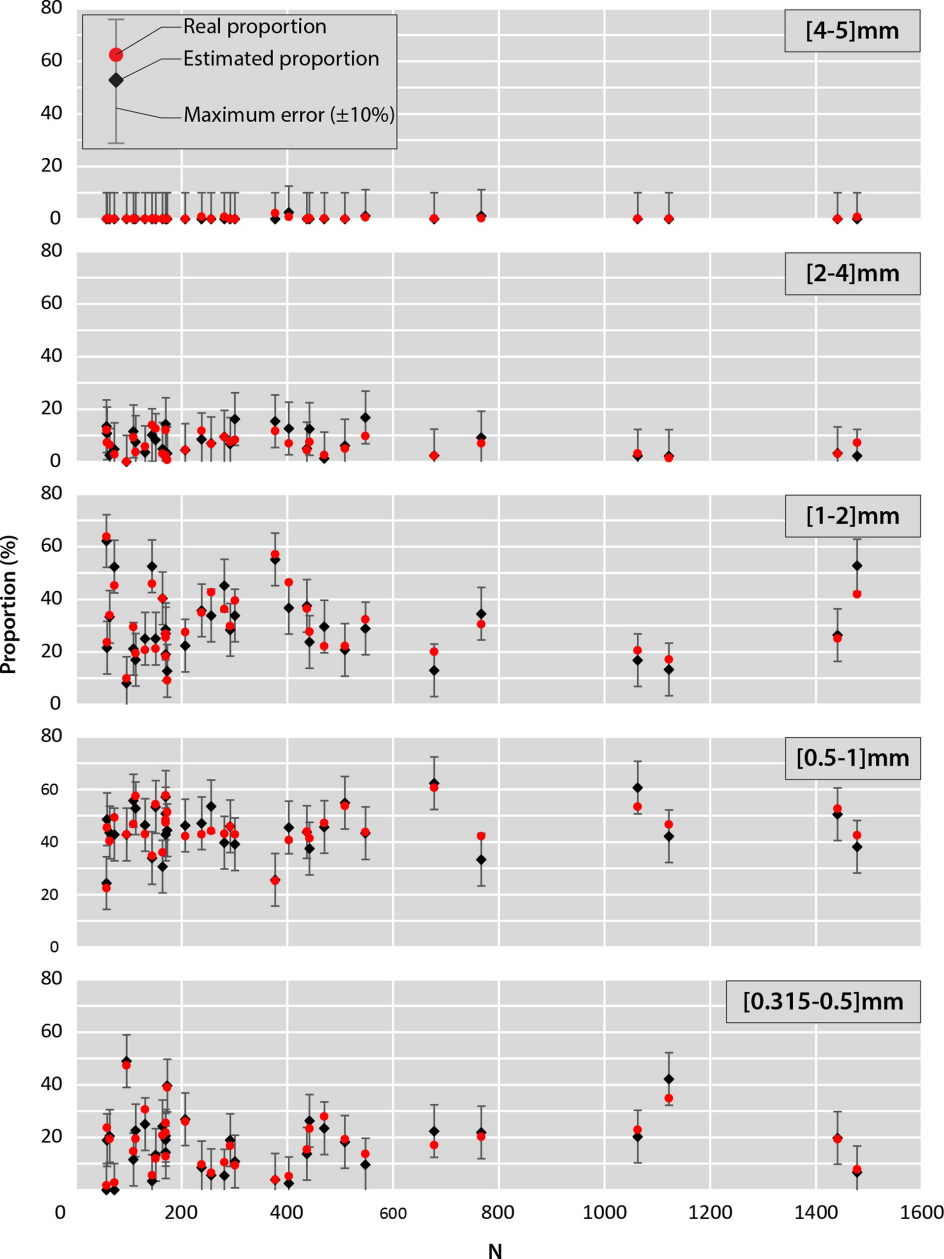
Variation of the size class proportions as the function of the manta population (N; number of microplastics per manta) and margin errors associated.

The results show that some size classes are better represented than others. Thus, overall class [0.5–1] mm represents between 40 and 60% of the collected microplastics. On the contrary, classes [2–4] and [4–5] mm are underrepresented with generally less than 10 to 15% of the collected microplastics. Classes [0.315–0.5] and [1–2] mm are intermediate with average proportions in the order of 17 and 30% respectively. However, variations of the estimated proportion can be noted from one manta to another. This implies that the distribution of microplastic size depends of the location in the Mediterranean Sea. In this case, these variations of the estimated proportion do not appear to be related to the number of particles collected but related to the location. As we can see on Fig 5, the estimated proportion suffered very little variation with the growth of the number of particles. The analysis of the remaining samples and the linking of these results with the metadata acquired during the campaign could highlight factors impacting the granulometric distribution of microplastics in the Mediterranean Sea.

#### Step 4: Comparison of the proportions

When comparing real proportions and those obtained after random drawing 99.4% of the estimates have a ε_a_ <10% and calculations of the p-value show no statistical difference between real and estimated proportions for 41 mantas (p-value≥0.05). The only significant difference is obtained for the manta with the highest number of microplastics (p-value <0.05). On average the difference between the real proportion and the estimated proportion is 2.6 ± 2.4% so generally far below 10%.

### The manta by manta approach: The example of the polymer type

This protocol can also be used to study the proportions of different types of polymers that can be observed in the samples. The choice of the number of microplastics to be studied per manta being strictly identical to in point 4.2, only steps 2–3 and 4 will be detailed here. Since the comparison of the results with the real proportions of the manta involves the analysis of all the collected microplastics, the demonstration will be performed on the M23 and M14 manta.

#### Steps 2–3: Proportion and confidence interval

**Fig 6:**
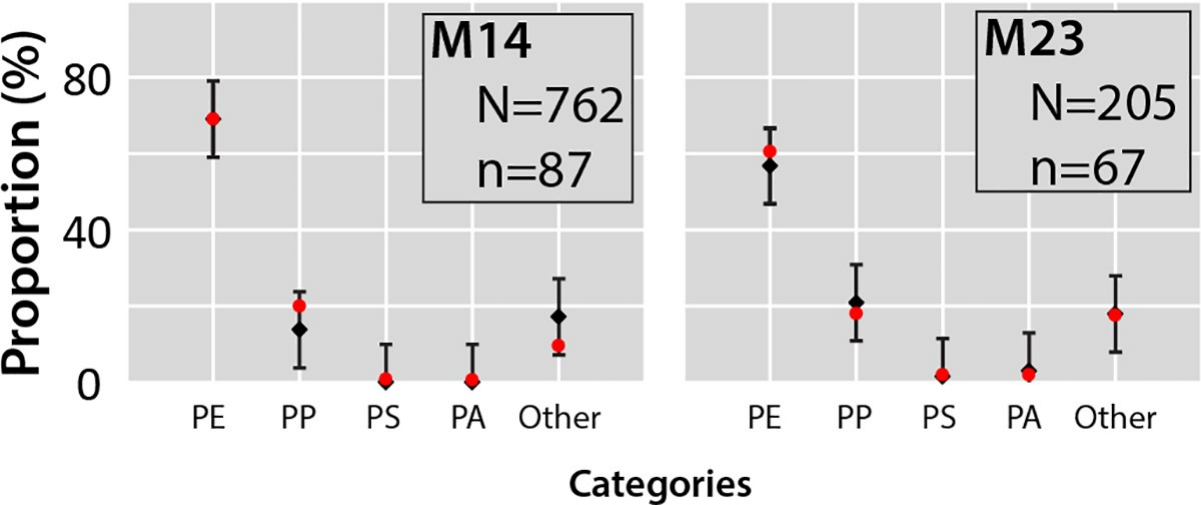
Real and estimated proportions of different polymers determined based on microplastic FTIR spectra. Real and estimated proportions are very similar and do not statistically differ.

Analysis of the FTIR spectra of microplastics of two mantas (M14 and M23) shows that these samples consist mainly of polyethylene and polypropylene (Fig 6). Other polymers, such as polyamide and polystyrene, account for less than 5% in M14 and M23 mantas. Unclear spectra, classified as “other”, account for about 17% of all particles. These results are very similar to those already observed in the Mediterranean Sea [24]. These results are also similar to those from Brest (France) [13] and Tamar Estuary (United Kingdom) [47], but with less polystyrene particles. This difference may be due to a greater distance offshore. The remaining manta samples will now be studied using the statistical approach to characterize the chemical nature of microplastics present in the surface layer of the east basin of the Mediterranean Sea.

#### Step 4: Comparison of the proportions

The real and estimated proportions are very similar and the maximum ε_a_ is 7.7% for M14 and 3.8% for M23, therefore below the fixed maximum error of 10%. Furthermore, based on p-values, the estimated proportions do not differ statistically from the real proportions. This approach makes possible to consider the generalization of spectrometric analysis in the case of campaigns with a large number of samples. In the case of this study, only two mantas have been analyzed so far in order to conduct this preliminary study. Thanks to this protocol, the other samples can now be analyzed.

## Conclusion

In highly contaminated environments, the number of microplastics collected by manta can be very high with values ranging from hundreds to thousands of particles as in the Mediterranean Sea [24]. Under such conditions, microplastic analysis can be very tedious, repetitive and time consuming. Statistical methods, like the one used here, are commonly used to conduct representative sampling of given populations. The protocol of analysis proposed here, which in some cases significantly reduces the number of microplastics to analyze, is an efficient way to help solve this problem. Thus, in the case of a sampling campaign, the results obtained by analyzing 400 to 3,000 randomly selected microplastics make it possible to provide a representative view of the microplastics of the area sampled, with an accuracy ranging from 2 to 5%. This methodology is relevant to help researchers to analyze microplastics collected during study campaigns on the scale of a sea. It can be used even for small areas of soil, sediment or beach with lower overall concentration levels of microplastics. This approach also opens the possibility to study large samples with successive methods (ex. FT-IR, RAMAN, SEM). In such cases, where the total number of measurements increases rapidly, the statistical approach can be applied to reduce the number of manipulations while controlling the error within acceptable limits. It is therefore likely that in the relatively near future, research programs will systematically attempt to describe the microplastic pollution using different descriptors. This will involve multiple analyses, which will be difficult, if not impossible, on oversized microplastics samples. The use of this statistical approach by reducing the number of microplastics required for analysis is a very robust and efficient tool.

## Supporting information

S1 File Detailed version of the statistical protocol. (DOCX)

## References

[1] Barboza LG, Gimenez BC (2015) Microplastics in the marine environment: Current trends and future perspectives. Mar Pollut Bull 97: 5–12. 10.1016/j.marpolbul.2015.06.008 26072046

[2] Dussud C, Meistertzheim AL, Conan P, Pujo-Pay M, George M, et al (2018) Evidence of niche partitioning among bacteria living on plastics, organic particles and surrounding seawaters. Environmental Pollution 236: 807–816. 10.1016/j.envpol.2017.12.027 29459335

[3] Qiu Q, Tan Z, Wang J, Peng J, Li M, et al (2016) Extraction, enumeration and identification methods for monitoring microplastics in the environment. Estuarine, Coastal and Shelf Science 176: 102–109.

[4] Ng KL, Obbard JP (2006) Prevalence of microplastics in Singapore’s coastal marine environment. Marine Pollution Bulletin 52: 761–767. 10.1016/j.marpolbul.2005.11.017 16388828

[5] Frias JP, Gago J, Otero V, Sobral P (2016) Microplastics in coastal sediments from Southern Portuguese shelf waters. Marine Environmental Research 114: 24–30. 10.1016/j.marenvres.2015.12.006 26748246

[6] Eriksen M, Mason S, Wilson S, Box C, Zellers A, et al (2013) Microplastic pollution in the surface waters of the Laurentian Great Lakes. Marine Pollution Bulletin 77: 177–182. 24449922 10.1016/j.marpolbul.2013.10.007

[7] Guven O, Gokdag K, Jovanovic B, Kideys AE (2017) Microplastic litter composition of the Turkish territorial waters of the Mediterranean Sea, and its occurrence in the gastrointestinal tract of fish. Environ Pollut 223: 286–294. 10.1016/j.envpol.2017.01.025 28117186

[8] Akhbarizadeh R, Moore F, Keshavarzi B, Moeinpour A (2017) Microplastics and potentially toxic elements in coastal sediments of Iran's main oil terminal (Khark Island). Environ Pollut 220: 720–731. 10.1016/j.envpol.2016.10.038 27769770

[9] Cózar A, Martí E, Duarte CM, García-de-Lomas J, van Sebille E, et al (2017) The Arctic Ocean as a dead end for floating plastics in the North Atlantic branch of the Thermohaline Circulation. Science Advances 3. 10.1126/sciadv.1600582 PMC5397136 28439534

[10] Dris R, Gasperi J, Mirande C, Mandin C, Guerrouache M, et al (2017) A first overview of textile fibers, including microplastics, in indoor and outdoor environments. Environ Pollut 221: 453–458. 10.1016/j.envpol.2016.12.013 27989388

[11] Kedzierski M, D'Almeida M, Magueresse A, Le Grand A, Duval H, et al (2018) Threat of plastic ageing in marine environment. Adsorption/desorption of micropollutants. Mar Pollut Bull 127: 684–694. 10.1016/j.marpolbul.2017.12.059 29475712

[12] Song YK, Hong SH, Jang M, Han GM, Rani M, et al (2015) A comparison of microscopic and spectroscopic identification methods for analysis of microplastics in environmental samples. Mar Pollut Bull 93: 202–209. 10.1016/j.marpolbul.2015.01.015 25682567

[13] Frère L, Paul-Pont I, Moreau J, Soudant P, Lambert C, et al (2016) A semi-automated Raman micro-spectroscopy method for morphological and chemical characterizations of microplastic litter. Mar Pollut Bull 113: 461–468. 10.1016/j.marpolbul.2016.10.051 27837909

[14] Dümichen E, Barthel AK, Braun U, Bannick CG, Brand K, et al (2015) Analysis of polyethylene microplastics in environmental samples, using a thermal decomposition method. Water Res 85: 451–457. 10.1016/j.watres.2015.09.002 26376022

[15] Majewsky M, Bitter H, Eiche E, Horn H (2016) Determination of microplastic polyethylene (PE) and polypropylene (PP) in environmental samples using thermal analysis (TGA-DSC). Sci Total Environ 568: 507–511. 10.1016/j.scitotenv.2016.06.017 27333470

[16] Brennecke D, Duarte B, Paiva F, Caçador I, Canning-Clode J (2016) Microplastics as vector for heavy metal contamination from the marine environment. Estuarine, Coastal and Shelf Science 178: 189–195.

[17] Boucher C, Morin M, Bendell LI (2016) The influence of cosmetic microbeads on the sorptive behavior of cadmium and lead within intertidal sediments: A laboratory study. Regional Studies in Marine Science 3: 1–7.

[18] Bakir A, Rowland SJ, Thompson RC (2012) Competitive sorption of persistent organic pollutants onto microplastics in the marine environment. Marine Pollution Bulletin 64: 2782–2789. 10.1016/j.marpolbul.2012.09.010 23044032

[19] Bakir A, Rowland SJ, Thompson RC (2014) Enhanced desorption of persistent organic pollutants from microplastics under simulated physiological conditions. Environmental Pollution 185: 16–23. 10.1016/j.envpol.2013.10.007 24212067

[20] Bakir A, O'Connor IA, Rowland SJ, Hendriks AJ, Thompson RC (2016) Relative importance of microplastics as a pathway for the transfer of hydrophobic organic chemicals to marine life. Environmental Pollution 219: 56–65. 10.1016/j.envpol.2016.09.046 27661728

[21] Zettler ER, Mincer TJ, Amaral-Zettler LA (2013) Life in the "plastisphere": Microbial communities on plastic marine debris. Environmental Science and Technology 47: 7137–7146. 10.1021/es401288x 23745679

[22] Amaral-Zettler LA, Zettler ER, Slikas B, Boyd GD, Melvin DW, et al (2015) The biogeography of the Plastisphere: implications for policy. Frontiers in Ecology and the Environment 13: 541–546.

[23] Frère L, Maignien L, Chalopin M, Huvet A, Rinnert E, et al (2018) Microplastic bacterial communities in the Bay of Brest: Influence of polymer type and size. Environmental Pollution 242: 614–625. 10.1016/j.envpol.2018.07.023 30014939

[24] Pedrotti ML, Petit S, Elineau A, Bruzaud S, Crebassa JC, et al (2016) Changes in the Floating Plastic Pollution of the Mediterranean Sea in Relation to the Distance to Land. PLoS One 11: e0161581 10.1371/journal.pone.0161581 27556233 PMC4996504

[25] Rocha-Santos T, Duarte AC (2015) A critical overview of the analytical approaches to the occurrence, the fate and the behavior of microplastics in the environment. TrAC Trends in Analytical Chemistry 65: 47–53.

[26] Eriksen M, Maximenko N, Thiel M, Cummins A, Lattin G, et al (2013) Plastic pollution in the South Pacific subtropical gyre. Marine Pollution Bulletin 68: 71–76. 10.1016/j.marpolbul.2012.12.021 23324543

[27] Eriksen M, Lebreton LC, Carson HS, Thiel M, Moore CJ, et al (2014) Plastic Pollution in the World's Oceans: More than 5 Trillion Plastic Pieces Weighing over 250,000 Tons Afloat at Sea. PLoS One 9: e111913 10.1371/journal.pone.0111913 25494041 PMC4262196

[28] Moore CJ, Moore SL, Leecaster MK, Weisberg SB (2001) A comparison of plastic and plankton in the North Pacific central gyre. Marine Pollution Bulletin 42: 1297–1300. 11827116 10.1016/s0025-326x(01)00114-x

[29] Law KL, Morét-Ferguson S, Maximenko NA, Proskurowski G, Peacock EE, et al (2010) Plastic Accumulation in the North Atlantic Subtropical Gyre. Science 329: 1185–1188. 10.1126/science.1192321 20724586

[30] Lebreton LCM, Greer SD, Borrero JC (2012) Numerical modelling of floating debris in the world’s oceans. Marine Pollution Bulletin 64: 653–661. 10.1016/j.marpolbul.2011.10.027 22264500

[31] van Sebille E, Wilcox C, Lebreton L, Maximenko N, Hardesty BD, et al (2015) A global inventory of small floating plastic debris. Environmental Research Letters 10: 124006.

[32] Cózar A, Sanz-Martín M, Martí E, González-Gordillo JI, Ubeda B, et al (2015) Plastic Accumulation in the Mediterranean Sea. PLOS ONE 10: e0121762 10.1371/journal.pone.0121762 25831129 PMC4382178

[33] Schlitzer R (2015) Data Analysis and Visualization with Ocean Data View. Canadian Meteorological and Oceanographic Society 43: 9–13.

[34] Hidalgo-Ruz V, Gutow L, Thompson RC, Thiel M (2012) Microplastics in the Marine Environment: A Review of the Methods Used for Identification and Quantification. Environmental Science & Technology 46: 3060–3075. 22321064 10.1021/es2031505

[35] Moore CJ, Moore SL, Weisberg SB, Lattin GL, Zellers AF (2002) A comparison of neustonic plastic and zooplankton abundance in southern California’s coastal waters. Marine Pollution Bulletin 44: 1035–1038. 12474963 10.1016/s0025-326x(02)00150-9

[36] Löder MGJ, Gerdts G (2015) Methodology Used for the Detection and Identification of Microplastics—A Critical Appraisal In: Bergmann M, Gutow L, Klages M, editors. Marine Anthropogenic Litter. Cham: Springer International Publishing pp. 201–227.

[37] Kanhai DK, Officer R, Lyashevska O, Thompson RC, O'Connor I (2017) Microplastic abundance, distribution and composition along a latitudinal gradient in the Atlantic Ocean. Mar Pollut Bull 115: 307–314. 10.1016/j.marpolbul.2016.12.025 28007381

[38] Ogata Y, Takada H, Mizukawa K, Hirai H, Iwasa S, et al (2009) International Pellet Watch: Global monitoring of persistent organic pollutants (POPs) in coastal waters. 1. Initial phase data on PCBs, DDTs, and HCHs. Marine Pollution Bulletin 58: 1437–1446. 10.1016/j.marpolbul.2009.06.014 19635625

[39] Antunes JC, Frias JGL, Micaelo AC, Sobral P (2013) Resin pellets from beaches of the Portuguese coast and adsorbed persistent organic pollutants. Estuarine, Coastal and Shelf Science 130: 62–69.

[40] Bakir A, Rowland SJ, Thompson RC (2014) Transport of persistent organic pollutants by microplastics in estuarine conditions. Estuarine, Coastal and Shelf Science 140: 14–21.

[41] Sussarellu R, Suquet M, Thomas Y, Lambert C, Fabioux C, et al (2016) Oyster reproduction is affected by exposure to polystyrene microplastics. Proc Natl Acad Sci U S A 113: 2430–2435. 10.1073/pnas.1519019113 26831072 PMC4780615

[42] Imhof HK, Laforsch C, Wiesheu AC, Schmid J, Anger PM, et al (2016) Pigments and plastic in limnetic ecosystems: A qualitative and quantitative study on microparticles of different size classes. Water Res 98: 64–74. 10.1016/j.watres.2016.03.015 27082693

[43] Turner A (2016) Heavy metals, metalloids and other hazardous elements in marine plastic litter. Marine Pollution Bulletin 111: 136–142. 10.1016/j.marpolbul.2016.07.020 27452160

[44] Wang J, Peng J, Tan Z, Gao Y, Zhan Z, et al (2017) Microplastics in the surface sediments from the Beijiang River littoral zone: Composition, abundance, surface textures and interaction with heavy metals. Chemosphere 171: 248–258. 10.1016/j.chemosphere.2016.12.074 28024210

[45] Lobelle D, Cunliffe M (2011) Early microbial biofilm formation on marine plastic debris. Marine Pollution Bulletin 62: 197–200. 10.1016/j.marpolbul.2010.10.013 21093883

[46] McCormick A, Hoellein TJ, Mason SA, Schluep J, Kelly JJ (2014) Microplastic is an Abundant and Distinct Microbial Habitat in an Urban River. Environmental Science & Technology 48: 11863–11871. 25230146 10.1021/es503610r

[47] Sadri SS, Thompson RC (2014) On the quantity and composition of floating plastic debris entering and leaving the Tamar Estuary, Southwest England. Marine Pollution Bulletin 81: 55–60. 10.1016/j.marpolbul.2014.02.020 24613232

