# Supplementary material for: Microplastics in Mediterranean Sea: A protocol to robustly assess contamination characteristics
Source: PLoS One. 2019 Feb 11;14(2):e0212088. doi: 10.1371/journal.pone.0212088 (PMC6370211; doi:10.1371/journal.pone.0212088)
Supplement: S1 File — (DOCX) [file pone.0212088.s001.docx]

**3.1. Statistical approach**

In a population (in this study, microplastics of the Mediterranean Sea), to estimate an unknown proportion $\pi\in\left[ 0;1 \right]$ of units possessing a characteristic $C$ (e.g. the chemical nature of a microplastic), an estimation of the parameter of a Bernouilli law is used. In this configuration, it is considered that each microplastic is randomly drawn and therefore

$$P\left( the microplastic possess the characteristic C \right)=\pi$$

or

$P(the microplastic does not possess the characteristic C)=1-\pi$.

3.1.1. Proportion estimator

The random variable X is defined by $X=1$ if the chosen unit has $C$ and $X=0$ otherwise. Knowing the distribution law of $\pi$, it was possible to construct a good estimator p for the proportion $\pi$ from a sample of size n. Note that coding 0 or 1 implies that $\sum_{i=1}^{n} x_{i}$ is the exact number of sample units possessing C and a succession of draws of X follows a Binomial law. So, an estimation p corresponds to $E\left[ X \right]$ and could be calculated by

$p=\frac{1}{n}\sum_{i=1}^{n} x_{i}$ (1)

For this estimator, since the distribution law of X is known, it was possible to construct a confidence interval for p. To construct this interval, two configurations had to be denoted. The first configuration, in which the global population size (N) is unknown, was used in the case of the Mediterranean Sea, since it is not possible to know the exact number of microplastics present. The second configuration was the case in which the population size (N) is known. The number (N) of microplastics collected manta by manta was known and the proportion would be estimated from a subsample of size (n). Based on these two configurations, a confidence interval could be given for each one.

3.1.2. Confidence interval

Once the estimator p of the proportion had been determined, the associated confidence interval had to be calculated. An estimator for the variability of X had to be constructed. Due to the Bernoulli law, the variability of X/n is known as

$$Var\left[ \frac{X}{n} \right]=\frac{p\left( 1-p \right)}{n}$$

Because it took a value between 0 or 1, p was considered the average of X. Using the Central limit theorem, it can be shown that the estimator of the proportion p followed normal law $\mathcal{N}(0,1)$. In the configuration where N is unknown, the confidence interval for the confidence level $\alpha$ is given by

$IC_{\frac{\alpha}{2}}=\left[ {p \pm u}_{1-\frac{\alpha}{2}}\sqrt{\frac{p\left( 1-p \right)}{n}} \right]$ (2)

with $u_{1-\frac{\alpha}{2}}$ the fractal of order $\alpha$ of the standardized normal law. It is common to take as degree of confidence of 95% (*i.e.* $\alpha=0.05$; $u_{1- \frac{\alpha}{2}}=1.96$). For the configuration in which the proportion was estimated when N is known, a confidence interval for confidence level $\alpha$ was given by

${IC}_{\frac{\alpha}{2}}=\left[ {p\pm u}_{1- \frac{\alpha}{2}}\sqrt{\frac{p\left( 1-p \right)}{n}*\frac{(N-n)}{(N-1)}} \right]$ (3)

with $u_{1-\frac{\alpha}{2}}$ the fractal of order $\alpha$ of the standardized normal law. The protocol presented below is based on this statistical approach.

**3.2. Protocols**

To estimate the proportion of microplastics with a characteristic C, two protocols corresponding to the two configurations were used, following the 4 steps described below. The first step was the estimation of the number of microplastics needed to obtain a given accuracy (*i.e.* the value measuring half width of the confidence interval). The second one was the estimation of the proportion. The third step consisted in giving the confidence interval of the estimated proportion. The last step described the tests used for comparing two proportions.

3.2.1. Step 1: number of microplastics

To be able to study the characteristics of the collected microplastics, it is first necessary to determine the minimum number of particles to be studied to reach a certain confidence level in the estimated proportion. In the statistical approach (3.1), it had been shown that accuracy depended on the proportion (p) and size of the sub-sample (n). In the first configuration, the difficulty was that p is unknown. Nevertheless, an upper bound can be found to the function $p(1-p)$ as it is maximum when $p=\frac{1}{2}$.

In the configuration in which the estimation of the proportion of microplastics possessing a characteristic C in the Mediterranean Sea was aimed at p can therefore be substituted by $\frac{1}{2}$. Thus, the accuracy (ε) at the degree of confidence 1 - α of a proportion was calculated as:

${\varepsilon= u}_{1- \frac{\alpha}{2}}\sqrt{\frac{1}{4n}}$

Then, the number of microplastics that had to be randomly drawn was calculated for three classic values of accuracy: 5%, 2.5% and 1%. To this end, the following equation was used:

$n=\frac{{{(u}_{1- \frac{\alpha}{2}})}^{2}}{4\varepsilon^{2}}$ (4)

In the configuration in which the aim was to estimate the proportion of microplastics possessing a characteristic C in a manta, N is known. Substituting p by $\frac{1}{2}$, the accuracy (ε) of a proportion, at the degree of confidence 1 - α, was calculated as:

$u_{1- \frac{\alpha}{2}}\sqrt{\frac{1}{4n}*\frac{(N-n)}{(N-1)}}$

For a given error, the number of microplastics in the sampled population was obtained as follows:

$n=\frac{\frac{1}{4}+\frac{\varepsilon^{2}}{\left( u_{1- \frac{\alpha}{2}} \right)^{2}}}{\frac{\varepsilon^{2}}{\left( u_{1- \frac{\alpha}{2}} \right)^{2}}+\frac{1}{4N}}$ (5)

From the equation (4) and (5), it was then possible to calculate, the number of microplastics to analyze for a given accuracy.

3.2.2. Step 2-3: Proportion and confidence interval

Once the minimum number of particles to be analyzed had been determined, microplastics can be subsampled and analyzed. The proportion of microplastics with the characteristic C had to be calculated using equation (1). Then, the confidence interval of order α was calculated. To construct the confidence interval, the equation (2) was used for the first configuration (Mediterranean Sea) and the equation (3) for the second configuration (estimation manta by manta).

3.2.3. Step 4: comparison of the proportions

Now that the proportions were estimated and the confidence intervals defined. The proportions (p) could be compared with a theoretical value or with the proportions from two different samples. So, to assess the significance of the difference between the values of the proportions (p) obtained after sub-sampling and those of references (π), two statistical indicators are calculated: the absolute error and the test of equal or given proportions. The absolute error (ε_a_) is the absolute difference between a proportion of microplastics possessing the characteristic C (π) and its estimated proportion in the population (p). The absolute error is given by:

$\varepsilon_{a}=\left| \pi-p \right|$

The test of equal proportions, on the other hand, allows the comparison between an observed proportion and a theoretical proportion. The aim of this test is to verify if, in a population P (here the microplastics in the Mediterranean Sea), a proportion differs or not from a reference value. The use of this test is based on a test value (z)

$$z=\frac{p-\pi}{\sqrt{\frac{\pi\left( 1-\pi\right)}{n}}}$$

which can be approximated by a normal law. In this case, the hypothesis $H_{0}:p= \pi$ was tested. If $|z|>u_{1-\frac{\alpha}{2}}$, then the hypothesis was rejected.

To compare two proportions, a statistical test was used. Thanks to the central limit theorem, it is known that the distribution of a proportion is a normal distribution centered on the true value of the proportion to be estimated. To compare the estimated proportions on two independent samples, the $\chi^{2}$ test is used because of the normal distribution of the proportions. Under H0, the $\chi^{2}$ test statistic is calculated as

$$\chi^{2}=\sum_{i,j} \frac{\left( N_{\mathrm{ij}}-T_{\mathrm{ij}} \right)^{2}}{T_{\mathrm{ij}}}$$
